# 11,12 Epoxyeicosatrienoic Acid Rescues Deteriorated Wound Healing in Diabetes

**DOI:** 10.3390/ijms222111664

**Published:** 2021-10-28

**Authors:** Katharina Sommer, Heike Jakob, Caroline Reiche, Dirk Henrich, Jasmina Sterz, Johannes Frank, Ingo Marzi, Anna Lena Sander

**Affiliations:** 1 Department of Trauma, Hand and Reconstructive Surgery, Hospital of the Johann Wolfgang Goethe-University, 60596 Frankfurt am Main, Germany; dirk.henrich@kgu.de (D.H.); jasmina.sterz@kgu.de (J.S.); johannes.frank@kgu.de (J.F.); ingo.marzi@kgu.de (I.M.); annalena.sander@kgu.de (A.L.S.); 2 Department of Trauma, Hand and Reconstructive Surgery, Marienhausklinik St. Josef Kohlhof, 66539 Neunkirchen, Germany; heike.jakob@marienhaus.de; 3 Department of Surgery, Hospital Bad Soden, 65812 Bad Soden, Germany; carolinereiche@googlemail.com

**Keywords:** wound repair, diabetes, EET, neovascularization, proliferation, inflammatory reaction

## Abstract

Epoxyeicosatrienoic acids (EET) facilitate regeneration in different tissues, and their benefit in dermal wound healing has been proven under normal conditions. In this study, we investigated the effect of 11,12 EET on dermal wound healing in diabetes. We induced diabetes by i.p. injection of streptozotocin 2 weeks prior to wound creation on the dorsal side of the mouse ear. 11,12 EET was applied every second day on the wound, whereas the control groups received only solvent. Epithelialization was monitored every second day *intravitally* up to wound closure. Wounds were stained for VEGF, CD31, TGF-β, TNF-α, SDF-1α, NF-κB, and Ki-67, and fibroblasts were counted after hematoxylin-eosin stain on days 3, 6, 9, and 16 after wounding. After induction of diabetes, wounds closed on day 13.00 ± 2.20 standard deviation (SD). Local 11,12 ETT application improved wound closure significantly to day 8.40 ± 1.39 SD. EET treatment enhanced VEGF and CD31 expression in wounds on day 3. It also seemed to raise TNF-α level on all days investigated as well as TGF-β level on days 3 and 6. A decrease in NF-κB could be observed on days 9 and 16 after EET application. The latter findings were not significant. SDF-1α expression was not influenced by EET application, and Ki-67 was significantly less in the EET group on day 9 after EET application. The number of fibroblasts was significantly increased on day 9 after the 11,12 EET application. 11,12 EET improve deteriorated wound healing in diabetes by enhancing neoangiogenesis, especially in the early phase of wound healing. Furthermore, they contribute to the dissolution of the initial inflammatory reaction, allowing the crucial transition from the inflammatory to proliferative phase in wound healing.

## 1. Introduction

Chronic ulcers caused by diabetes mellitus still impose an immense problem in modern medicine. Despite improved therapies, more than 50% of diabetic ulcers tend to fail to heal, often resulting in limb amputation (Liu und Velazquez 2008). Thus, the improvement of wound healing in diabetes is still of broad interest.

Recently, several studies have focused on promoting angiogenesis to improve the closure of diabetic ulcers as they fail in healing, among other things, because of impaired angiogenic response [1,2]. Moreover, decreased growth factor production contributes to poor healing in diabetes [3,4].

Among these growth factors, vascular endothelial growth factor (VEGF) is one of the most potent pro-angiogenic signaling proteins in wound healing. It promotes endothelial cell activation and migration [5]. Enhanced VEGF expression induces an augmentation in angiogenesis that can be evaluated by CD31 expression, causing a faster wound closure [1]. Patients with chronic wounds especially display a decreased VEGFR-2 expression that causes reduced angiogenesis [6].

Besides this deficiency, diabetic wounds also show a shortage in the production of tumor necrosis factor-α (TNF-α), making wounds more susceptible to infection in the initial phase of wound healing [7]. In contrast to this, chronic diabetic ulcers exhibit a higher expression of TNF-α than wounds of healthy individuals in the later stages of wound healing, demonstrating that chronic wounds fail to resolve inflammation and are unable to switch to a proliferative state [8]. This is supported by the fact that high concentrations of TNF-α prevent granulation tissue formation and suppress the function of transforming growth factor-β (TGF-β) [9,10,11,12]. TGF-β1 is essential in wound healing as it initiates inflammation as well as granulation tissue formation [13]. It is also known that TGF-β signaling is impaired in diabetic wounds inhibiting cell migration and myofibroblast formation [14].

Fibroblasts themselves play a major role throughout the whole wound healing process [15]. In chronic wounds, fibroblasts exhibit early senescence, diminished proliferation, and an altered pattern of cytokine release when compared with normal wound fibroblasts [16,17]. 

High glucose level also influences the proliferative ability of keratinocytes negatively in vitro [18]. It also inhibits endothelial cell migration by enhanced nuclear factor κB (NF-κB) signaling, hindering wound healing [19]. Furthermore, wounds of diabetic mice exhibit a decreased expression of stromal cell-derived factor-1α (SDF-1α), and the elevation of SDF-1α expression is able to ameliorate this deteriorated wound healing [20,21]. SDF-1α is essential for the attraction of stem and progenitor cells to the wound site [22]. These cells enhance repair by their cytokine production [22].

Thus, improving growth factor production and angiogenesis might be a worthwhile strategy to ameliorate deteriorated wound healing. 

Epoxyeicosatrienoic acids (EET) are arachidonic acid-derived lipid mediators that can regulate inflammation, angiogenesis, and vascular tone [23]. In previous works, EET have been a promising tool to improve wound healing under normal conditions as well as in ischemic wounds [24,25]. Especially in ischemia, EET enhance VEGF expression in wounds, thus promoting angiogenesis [25,26].

Moreover, EET promote SDF-1α expression by cytokine production [27]. SDF-1α also enhances keratinocyte proliferation and migration in vitro, which could partly explain its positive effect on wound repair [27]. 

EET are also able to protect against the effects of TNF-α, thus diminishing organ damage in a rat model of lung injury [28]. Furthermore, EET can enhance tissue growth and regeneration in different organs and have anti-inflammatory properties, as well as enhancing vasodilation, cellular proliferation, and migration [23]. They also have been shown to enhance the expression of fibroblast growth factor 2 (FGF2), which raises fibroblast proliferation and ameliorates angiogenesis [29,30].

In the following study, we evaluated local EET application in wound healing in diabetic homozygous, fully immunocompetent hairless mice and analyzed the effects on inflammation and neovascularization in wounds.

## 2. Results

### 2.1. Wound Closure and Reepithelialization Process during Wound Healing

We evaluated the effect of 11,12 EET on wounds of diabetic mice by measuring the day of wound closure and the process of reepithelialization throughout the wound healing process. For the latter purpose, wound area was measured every second day after wounding of the ear.

**Figure 1:**
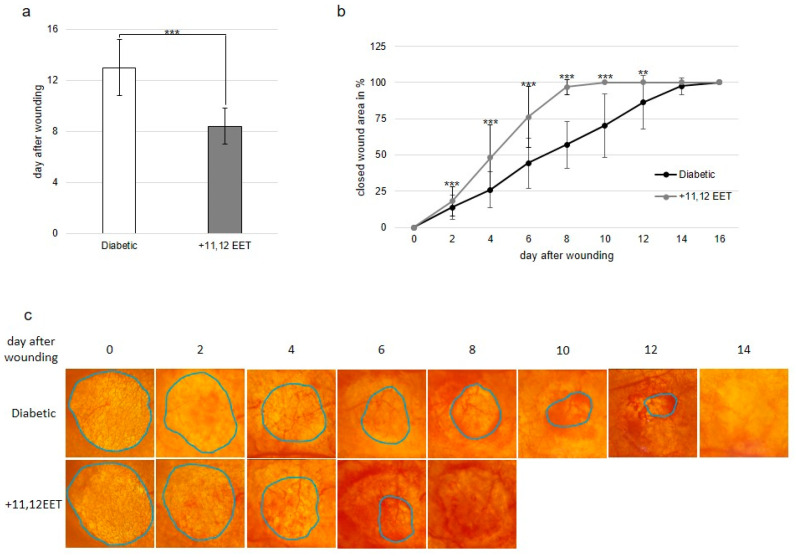
(**a**) Day of wound closure. Comparison between non-treated and 11,12 EET-treated wounds in diabetic mice. (**b**) Percentage of closed wound area from days 0–16. Comparison between wounds of diabetic animals and after treatment with 11,12 EET. (**c**) Representative pictures of wounds throughout the wound healing process in diabetic mice and after treatment with 11,12 EET. (Data are shown as mean ± SD; *n* = 20) ** *p* ≤ 0.01, *** *p* ≤ 0.001.

In diabetic control animals, wounds closed on day 13.0 ± 2.2 SD. Treatment with 11,12 EET accelerated wound closure significantly to 8.4 ± 1.4 SD days (Figure 1a). 

This result was also confirmed by the measurement of closing wound area in vivo throughout the wound healing process. Here, reepithelialization was accelerated after 11,12 EET treatment from day 2 to day 12 after wounding (Figure 1b). 

### 2.2. Evaluation of Local Neovascularization in Wounds

Granulation tissue formation and reepithelialization are dependent on the formation of new vessels. Improvement of neovascularization in diabetic wounds seems to be a promising target for the amelioration of healing. Thus, we evaluated CD31 and VEGF expression immunohistochemically as markers for neovascularization in diabetic wounds after treatment with 11,12 EET.

**Figure 2:**
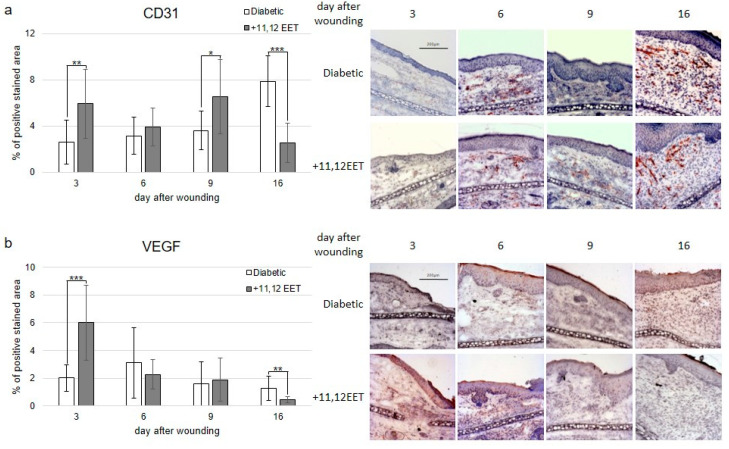
(**a**) Percentage of CD31-positive area on days 3, 6, 9, and 16 after wounding of diabetic mice without and after treatment with 11,12 EET; on the right, representative pictures of the staining. (**b**) Percentage of VEGF-positive cells on days 3, 6, 9, and 16 after wounding of diabetic mice without and after treatment with 11,12 EET; on the right, representative pictures of the staining. (Data are shown as mean ± SD; *n* = 10) * *p* ≤ 0.05, ** *p* ≤ 0.01, *** *p* ≤ 0.001.

Expression of CD31 was significantly increased on days 3 and 9 after treatment with 11,12 EET compared with diabetic control (Figure 2a). Local 11,12 EET treatment also enhanced expression of VEGF on day 3 after wounding (Figure 2b). In contrast to this finding, non-treated diabetic wounds exhibited an elevated level of CD31 and VEGF on day 16 (Figure 2a,b). In summary, local EET treatment enhances neovascularization as shown by CD31 and VEGF elevation in the early stages of wound healing in diabetic wounds. 

### 2.3. Evaluation of Local Inflammation Reaction in Wounds

The local inflammatory reaction to wounding was measured in diabetic wounds as well as after treatment with 11,12 EET by evaluating TNF-α and TGF-β expression. Expression of NF-κB was also evaluated immunohistochemically. Furthermore, the chemokine SDF-1α was stained in wounds as one of the main factors for the attraction of immune and progenitor cells. 

**Figure 3:**
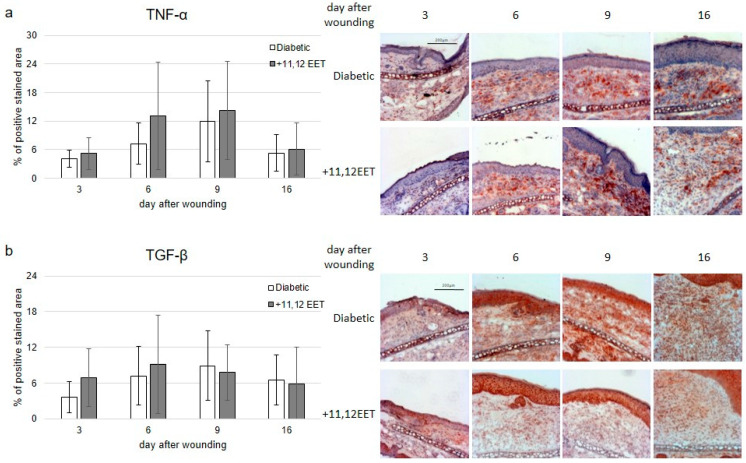
(**a**) Percentage of TNF-α-positive area on days 3, 6, 9, and 16 after wounding of diabetic mice without and after treatment with 11,12 EET; on the right, representative pictures of the staining. (**b**) Percentage of TGF-β-positive cells on days 3, 6, 9, and 16 after wounding of diabetic mice without and after treatment with 11,12 EET; on the right, representative pictures of the staining. (Data are shown as mean ± SD; *n* = 14).

Expression of TNF-α was not significantly changed by 11,12 EET application, though the level was higher than in diabetic control on day 6 after wounding (Figure 3a). 

TGF-β expression was elevated on days 3 and 6 after wounding compared with diabetic control, but not significantly (Figure 3b). 

**Figure 4:**
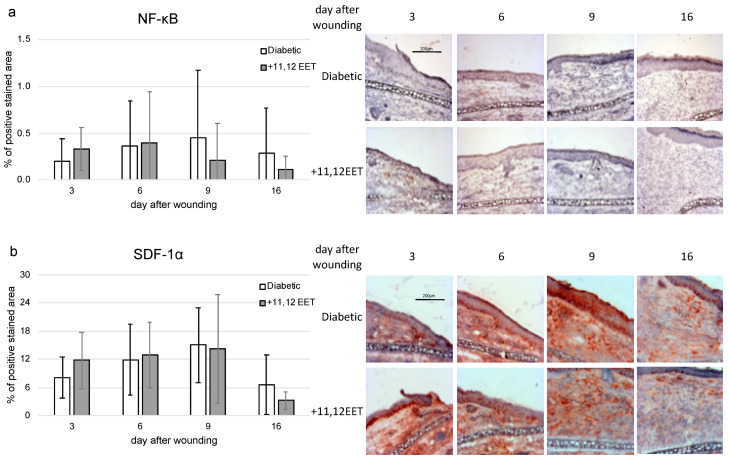
(**a**) Percentage of NF-κB-positive area on days 3, 6, 9, and 16 after wounding of diabetic mice without and after treatment with 11,12 EET; on the right, representative pictures of the staining (*n* = 10). (**b**) Percentage of SDF-1α-positive cells on days 3, 6, 9, and 16 after wounding of diabetic mice without and after treatment with 11,12 EET; on the right, representative pictures of the staining (*n* = 12). (Data are shown as mean ± SD).

11,12 EET application also slightly increased expression of NF-κB on day 3 after wounding (Figure 4a). In the later stages on days 9 and 16, levels seemed to be reduced after 11,12 EET treatment, though all these findings were not significant (Figure 4a). 

The level of SDF-1α seemed to be also increased on day 3, whereas on day 16 a reduced expression of SDF-1α in 11,12 EET-treated wounds could be noted, though both findings were not significant (Figure 4b).

### 2.4. Evaluation of Proliferation in Wounds

In our model, wound closure is mainly achieved by reepithelialization as the underlying cartilage of the mouse ear prevents wound contraction almost entirely. Therefore, the proliferation of keratinocytes is one of the main factors for faster wound closure in this model. Because diabetic wounds exhibited a significantly faster closure after treatment with EET, we evaluated the proliferation of keratinocytes on the wound edges by measuring Ki-67 expression.

**Figure 5:**
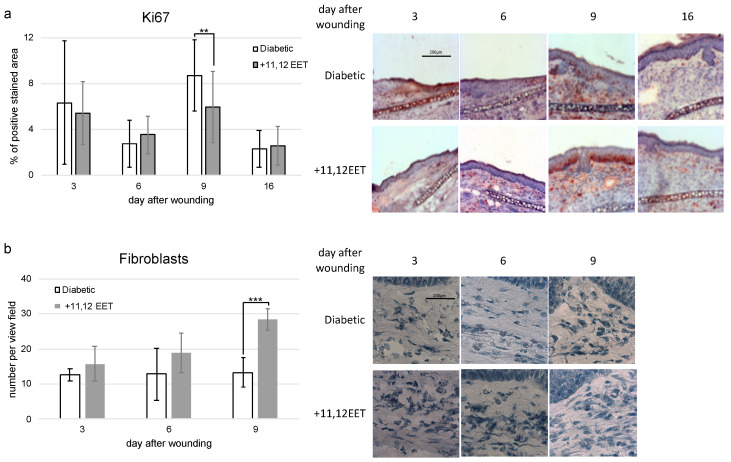
(**a**) Percentage of Ki-67-positive area on days 3, 6, 9, and 16 after wounding of diabetic mice without and after treatment with 11,12 EET; on the right, representative pictures of the staining. (**b**) Number of fibroblasts in the wound margin on days 2. 6, and 9 after wounding of diabetic mice without and after treatment with 11,12 EET; on the right, representative pictures of the staining. (Data are shown as mean ± SD; *n* = 14) ** *p* ≤ 0.01, *** *p* ≤ 0.001.

11,12 EET treatment did not change expression of Ki-67 on days 3 and 6 after wounding (Figure 5a). On day 9, the Ki-67 level was significantly higher in diabetic mice than in the treatment group (Figure 5a).

### 2.5. Evaluation of Number of Fibroblasts

As fibroblasts are essential for granulation tissue formation and consecutive wound healing, we counted the number of fibroblasts per field of view. 

Compared with control, the number of fibroblasts increased after 11,12 EET treatment on all days measured, though this finding was only significant for day 9 after wounding (Figure 5b). 

## 3. Discussion

In this project, we investigated the effect of 11,12 EET on impaired wound healing in diabetic mice. For this purpose, we used the wound model on the ear of the hairless mouse and induced diabetes by i.p. injection of streptozotocin 2 weeks prior to wounding. Animals with a blood glucose level higher than 200 mg/dl were considered diabetic and taken for wound healing experiments. The blood glucose level in healthy mice usually ranges around 100 mg/dl [31]. 

Local application of 11,12 EET significantly reduced the time to wound closure in diabetic animals from 13.00 days in control animals to 8.40 days in 11,12 EET-treated animals (Figure 1a). It also significantly decreased wound size from day 2 to day 12 after wounding (Figure 1b). Combined, these facts demonstrate that local application of 11,12 EET improves deteriorated wound healing in diabetes. 

In our previous experiments, healing of the same wound size on ears of untreated SKH-1 mice closed on day 8.0 [25], whereas wounds on ischemic ears closed on day 12.8. Thus, raising of blood glucose level by STZ seems to prolong wound healing to the same extent as ischemia in our model. Moreover, application of 11,12 EET nearly improves deteriorated wound healing to normal as normal healing takes place around day 8 and 11,12 EET-treated wounds of diabetic mice closed on day 8.4 [25]. 

To analyze the molecular mechanisms that caused amelioration of wound healing in diabetics caused by EET, we investigated the impact on neoangiogenesis as well as local inflammatory reaction and proliferation. 

Granulation tissue formation and reepithelialization are dependent on the formation of new vessels. Improvement of neovascularization in diabetic wounds seems to be a promising target for amelioration of healing as diabetic wounds are characterized by reduced angiogenesis [32]. 

EETs are known to enhance neovascularization by elevating VEGF expression [24]. Under diabetic conditions, 11,12 EET significantly enhanced VEGF expression in wounds on day 3 (Figure 2b). Accordingly, EET application also elevated CD31 levels in wounds on days 3 and 9 after wounding (Figure 2a). In diabetes, VEGF expression is usually decreased, and the application of VEGF-A alone can improve wound healing in diabetic mice [33,34]. In our hands, 11,12 EET were able to enhance VEGF as well as CD31 expression in diabetic mice. Therefore, enhancing neovascularization by augmentation of VEGF expression is a key mechanism to amelioration of wound healing by EET in diabetes. 

In contrast to this finding, non-treated diabetic wounds exhibited an elevated level of VEGF on day 16 after wounding compared with the 11,12 EET-treated counterparts. Accordingly, CD31 expression was decreased in 11,12 EET-treated diabetic wounds on day 16 as well. This enhanced expression of VEGF and CD31 in the latest point of time investigated in control wounds is likely linked to the longer wound closure in time in non-treated wounds as the healing process is still not finished in these wounds, and neoangiogenesis is still needed to be assigned for prolonged wound healing. The same effect of an increase in angiogenesis in wound healing in the control group at the latest point investigated was also observed in our earlier studies [25].

We further analyzed the effect of 11,12 EET on inflammatory reaction in wounds. The TNF-α level was not significantly influenced by 11,12 EET application in diabetic wounds, though it was slightly elevated on day 6 after wounding compared with the non-treated group. Thus, local 11,12 EET might be linked to a little elevated inflammatory reaction caused by 11,12 EET. TNF-α is known to enhance wound healing by stimulating fibroblast proliferation and promoting reepithelialization and neovascularization. It also enhances wound-breaking strength [35]. On the other hand, Maish et al. found that inhibition of TNF-α by a TNF-α binding protein improves the altered wound healing process in colonic anastomosis that is impaired by sepsis, encouraging the hypothesis that TNF-α is involved in the delay of wound healing [36,37]. Furthermore, diabetic wounds are marked by an elevated inflammation that causes a decreased vascularization as well as differentiation and proliferation of keratinocytes and fibroblasts [38]. Diabetic wounds also display an enhanced and prolonged expression of TNF-α [39]. 

These controversial effects might be linked to the level of increase in TNF-α as a moderate increase might lead to an amelioration in wound healing, whereas overwhelming expression of TNF-α hinders this process. This idea is supported by the fact, that a decreased level of TNF-α takes part in delayed wound healing in septic mice [25]. 

EET can protect against the pro-apoptotic effects of TNF-α, as Chen et al. showed in their animal model of lung injury. In this way, they were able to diminish organ damage [28]. This effect is independent of actual TNF-α concentration and mediated by modulating anti- and pro-apoptotic proteins by EET [40]. Therefore, the positive effect of 11,12 EET on wound healing in diabetes might not be linked to alteration of the expression of TNF-α in the model we used but could be linked to an alteration of its downstream mechanisms, which would be interesting to investigate in future studies. 

Furthermore, TNF-α is also known to suppress the function of TGF-β [12]. TGF-β is one of the most important factors for wound healing as it is a powerful chemoattractant for monocytes and macrophages, as well as fibroblasts [12]. Diminished signaling of TGF-β is involved in causing poor wound healing and inducing chronic ulcers in diabetes [14]. It promotes wound healing in normal and diabetic wounds [41]. It also induces angiogenesis and positively regulates ECM production by fibroblasts [42]. Enhanced expression of TGF-β also promotes faster epithelialization of wounds, though this role of the cytokine is yet to be discussed [43].

In our model, TGF-β expression was elevated on days 3 and 6 after wounding compared with non-treated control, though this was not significant (Figure 3b). Thus, the positive influence of EET on TGF-β might be another factor for the amelioration of wound healing in diabetes. 

Besides regulation of TGF-β, one of the effects of TNF-α is the activation of NF-κB [44]. Augmented NF-κB signaling deteriorates wound healing in diabetes by inhibiting endothelial cell migration [19]. Inhibition of NF-κB signaling in diabetic wounds enhances wound healing similarly to TGF-β treatment [45]. EET are able to suppress NF-κB activity by augmentation of IκB, the inhibitor of NF-κB [46]. Despite this fact, we found an increased expression of NF-κB on day 3 after wounding in the 11,12 EET-treated wounds (Figure 4a). On days 9 and 12, NF-κB expression was reduced to control (Figure 4a). Both findings were not significant. Therefore, there seems to be a higher initial inflammatory response after EET treatment, whereas in the later stages of wound healing, EET diminish NF-κB expression, thus allowing transition from the inflammatory to the proliferative phase. In this way, augmented initial inflammatory response and earlier attenuation of this process contribute to the amelioration of wound healing by EET in diabetes. 

In earlier studies, we found that EET can enhance SDF-1α expression during wound healing [24]. We also noticed an increase in SDF-1α on day 3 after wounding in the 11,12 EET-treated group (Figure 4b). On day 16 after wounding, SDF-1α was decreased compared with diabetic control, though both findings were not significant (Figure 4b). The homing factor SDF-1α attracts stem and progenitor cells to the side of tissue injury, which in turn stimulate repair by their cytokine production [22]. Furthermore, it can also enhance keratinocyte proliferation and migration in vitro, helping the process of reepithelialization [27]. Nevertheless, EET treatment did not significantly alter Ki-67 expression in wounds, though there seemed to be an elevated proliferation on day 9 after wounding in the control group. 

Furthermore, we investigated the effect of EET application on fibroblasts and found that local EET application raised the number of fibroblasts in wounds, though this finding was only significant on day 9 after wounding. Fibroblasts play a pivotal role throughout all phases of wound healing [47]. The enhanced number of fibroblasts we found in our investigation could be a result of the increase in FGF2 by EET application that has been shown previously, though further investigation needs to be carried out to support this notion [29].

To summarize, EET improve deteriorated wound healing in diabetes: first, by enhancing neoangiogenesis, especially in the early phase of wound healing; second, by contributing to dissolution of the initial inflammatory reaction allowing the crucial transition from inflammatory to proliferative phase in wound healing; and third, by enhanced homing via SDF-1α expression.

## 4. Material and Methods

For investigating wound healing under diabetic conditions, we induced diabetes mellitus by streptozotocin (STZ) in mice and combined this with the creation of a wound on the dorsal side of the ear. We analyzed epithelialization of the wounds in vivo in animals. Furthermore, neovascularization, inflammation, and proliferation were measured immunohistochemically by expression of CD31, VEGF, TNF-α, TGF-β, NF-κB, SDF-1α, and Ki-67 after the sacrifice of the animals on day 3, 6, 9, and 16 after wound creation. Number of fibroblasts was counted as well after hematoxylin staining.

### 4.1. Animals 

The animal experiments were conducted in conformity with the ethical guidelines of German law and approved by the Regierungspräsidium Darmstadt (Ethic Approval no. V54–19c20/15–F3/17). 

Male hairless SKH-1 mice (weight 25–35 g; age 8–10 weeks) were purchased from Charles River Laboratories (Sulzfeld, Germany). Animals were accommodated in separate cages at 24 °C with day/night intervals of 12 h/day in airflow regulated rooms and fed a balanced rodent diet with water ad libitum. For the surgical procedures as well as the wound measurements, the mice have anesthetized with an intraperitoneal (i.p.) injection of 100 µL solution containing 2.215 mg of ketamine and 0.175 mg of xylazine hydrochloride. At the set time points, animals were euthanized by cervical dislocation [48].

### 4.2. Induction of Diabetes

Diabetes in mice was induced by i.p. injection of STZ (Sigma-Aldrich, St. Louis, MO, USA) solved in 50 mM sodium acetate 1:10 weight adapted in a dose of 0.05 mg/g of body weight over 5 days as described earlier [31,49,50]. This procedure was carried out 2 weeks prior to the wounding of the ears. High blood glucose level was verified by puncturing of the tail vein with a blood glucose meter (Accu Chek Sensor and Accu Chek Sensor Comfort Pro-Teststreifen, Roche Diagnostics Deutschland GmbH, Mannheim, Germany). All animals with a blood glucose level exceeding 200 mg/dl were considered diabetic and were continued to use for wound healing experiments [31]. 

### 4.3. Wound Creation

Two weeks after induction of diabetes by STZ, circular wounds were created on the dorsum of both ears using a circular punch of 2.25 mm in diameter. A full thickness layer of ear skin was dissected down from the underlying cartilage as described previously [51]. Subsequently, a sheet of 2.5% methylcellulose in PBS with or without 11,12 EET was applied on the wound according to the treatment group. Sheets were produced by mixing 200 µL of 2.5% carboxymethylcellulose in PBS and adding 200 µL ethanol for control or 160 µL ethanol and 40 µL of 95% 11,12 EET. Then, the whole ear was covered with a bioadhesive occlusive dressing (Opsite; Smith and Nephew Medical Ltd., Tuttlingen, Germany) [48,52].

### 4.4. Wound Epithelialization and Wound Closure Measurements

Wounds on the dorsal side of the mouse ear allow direct measurement of epithelialization because contraction is prevented as the skin of the ear is firmly attached to the underlying cartilage. Thus, epithelialization could directly be measured in living animals through *intravital* microscopy.

Pictures of the wounds were taken on the day of wounding (day 0) and subsequently every second day thereafter. Wound area was measured on the photographs using computerized planimetry reflecting wound epithelialization. For the photographs, animals were anesthetized, and the ear was placed outstretched on an acrylic glass platform of a microscope (Carl Zeiss, Oberkochen, Germany). The image was taken with a low light camera (DXC-390P, 3CCD color video camera; Sony, Tokyo, Japan) and digitalized by a digital converter (ADVC-100; Canopus, Ruppach-Goldhausen, Germany). For calculation of the wound area, the wound margin on the images was traced utilizing the ImageJ software (http://rsb.info.nih.gov/ij/download.html (accessed on 20 June 2020)). The closed wound area was computed using the ratio of the wounded area at each time point divided by the original wound area at day 0. 

Day of wound closure was defined as the day of complete epithelialization. Analysis was carried out by an independent investigator blinded to the group setup.

### 4.5. Evaluation of Angiogenesis, Inflammatory Reaction, Proliferation, and Fibroblast Count in Wounds

Whole ears from killed mice were embedded in TissueTek (Sakura Finetek Europe, Zoeterwoude, The Netherlands) on days 3, 6, 9, and 16 after wounding and stored at −80 °C. Wounds were sliced to sections of 6 µm thickness and analyzed by immunohistochemical staining. 

The sections were fixed in acetone at −20 °C for 10 min and then blocked by 10 min 0.1% hydrogen peroxidase treatment. Afterward, they were stained with primary antibodies directed against VEGF and CD31 (ab7388, Abcam, Cambridge, UK) for evaluation of angiogenesis, TNF-α, TGF-β, SDF-1α (ab34719, ab66043, ab251117, Abcam, Cambridge, UK) and NF-κB (PAB0836, Abnova, Taipei City, Taiwan) for wound cytokine expression, and Ki-67 (ab15580, Abcam, Cambridge, UK) for proliferation. Incubation time for CD31, Ki-67, NF-κB, and TNF-α was 60 min at room temperature and for VEGF-A and SDF-1α overnight at +4 °C. Primary antibodies were detected by a suitable secondary antibody with Histofine (Nichirei Biosciences Inc., Tokyo, Japan) or HRP conjugated (Abcam, Cambridge, UK) and stained according to the guidelines of the manufacturer. Counterstaining was performed with hematoxylin. For fibroblast count, only HE staining was performed. 

Staining was then viewed at 100× magnification (Axio Observer; Carl Zeiss, Oberkochen, Germany). The microscope image was taken with a low-light digital camera (AxioCam; Carl Zeiss, Oberkochen, Germany). Images were analyzed using ImageJ software (https://imagej.nih.gov/ij/ (accessed on 20 June 2020)). The positive stained proportion of pixels viewed was standardized to the whole number of pixels of the image. This analysis was also conducted by an independent investigator. Fibroblasts were counted per field of view. For each wound, an average of three fields was counted.

### 4.6. Statistical Analysis

Data are displayed as mean ± SD. It was statistically analyzed with non-parametric Wilcoxon Mann–Whitney U Test by Bias 10.0 (Epsilon-Verlag, Darmstadt, Germany), comparing 11,12 EET-treated group and control at the same point of time. Results with *p* ≤ 0.05 were considered statistically significant. The number of samples examined is indicated by “*n*”.

## Data Availability

Data available on request. The data presented in this study are available on request from the corresponding author.

## Abbreviations

EET	epoxyeicosatrienoic acids
FGF2	fibroblast growth factor 2
SD	standard deviation
TNF-α	tumor necrosis factor-alpha
TGF-β	tumor growth factor-beta
VEGF	vascular endothelial growth factor
SDF-1α	stromal cell-derived factor 1α
STZ	streptozotocin
i.p.	intraperitoneal
